# Not all coping strategies are created equal: a mixed methods study exploring physicians' self reported coping strategies

**DOI:** 10.1186/1472-6963-10-208

**Published:** 2010-07-14

**Authors:** Jane B Lemaire, Jean E Wallace

**Affiliations:** 1 Faculty of Medicine, University of Calgary, Health Sciences Center, 3330 University Drive NW, Calgary, Alberta, T2N 4N1, Canada; 2 Department of Sociology, Faculty of Social Sciences, University of Calgary, 2500 University Drive NW, Calgary, Alberta, T2N 1N4, Canada

## Abstract

**Background:**

Physicians experience workplace stress and draw on different coping strategies. The primary goal of this paper is to use interview data to explore physicians' self reported coping strategies. In addition, questionnaire data is utilized to explore the degree to which the coping strategies are used and are associated with feelings of emotional exhaustion, a key symptom of burnout.

**Methods:**

This mixed methods study explores factors related to physician wellness within a large health region in Western Canada. This paper focuses on the coping strategies that physicians use in response to work-related stress. The qualitative component explores physicians' self reported coping strategies through open ended interviews of 42 physicians representing diverse medical specialties and settings (91% response rate). The major themes extracted from the qualitative interviews were used to construct 12 survey items that were included in the comprehensive quantitative questionnaire. Questionnaires were sent to all eligible physicians in the health region with 1178 completed surveys (40% response rate.) Questionnaire items were used to measure how often physicians draw on the various coping strategies. Feelings of burnout were also measured in the survey by 5 items from the Emotional Exhaustion subscale of the revised Maslach Burnout Inventory.

**Results:**

Major themes identified from the interviews include coping strategies used at work (e.g., working through stress, talking with co-workers, taking a time out, using humor) and after work (e.g., exercise, quiet time, spending time with family). Analysis of the questionnaire data showed three often used workplace coping strategies were positively correlated with feeling emotionally exhausted (i.e., keeping stress to oneself (r = .23), concentrating on what to do next (r = .16), and going on as if nothing happened (r = .07)). Some less often used workplace coping strategies (e.g., taking a time out) and all those used after work were negatively correlated with frequency of emotional exhaustion.

**Conclusions:**

Physicians' self reported coping strategies are not all created equal in terms of frequency of use and correlation with feeling emotionally exhausted from one's work. This knowledge may be integrated into practical physician stress reduction interventions.

## Background

Physicians regularly encounter stress while engaged in their professional activities [1-8] and likely utilize a variety of coping strategies to deal with their work stressors. Excessive stress may lead to burnout, a condition that has been associated with significant personal consequences for physicians (e.g. substance abuse and depression) and the health care system (e.g. poor quality of patient care and higher absenteeism and turnover rates) [3]. Coping strategies generally refer to behavioral and psychological efforts that are used to deal with stress. In previous quantitative survey studies of physician wellness, researchers have measured how often physicians use certain coping strategies using questionnaire items that rely on established coping measures from the general psychology literature [2,8,9]. For example, a questionnaire based study of general practitioners by King et al identified the most frequently used coping strategies as active coping, planning, restraint and acceptance, and the least frequently used as religion, denial, alcohol/drug use, and humor [9]. Another study of orthopedic surgery staff physicians described exercise, alcohol/drugs, religion, and talking with colleagues as infrequently used coping strategies, with more frequent use of strategies such as talking things over with family and friends [2]. There are few qualitative studies exploring how physicians themselves describe their coping strategies. One such study by Weiner et al undertook a thematic analysis of one final open ended question on a survey asking how physicians solve dilemmas related to their physical, emotional and spiritual well-being. They identified several primary wellness-promotion practice themes which included using relationships, religion or spirituality, self-care, and different approaches to life to cope with stress [10]. The primary objective of our mixed methods study is to explore how a sample of physicians describe their personal coping strategies for dealing with work related stress, both while they are at work and after leaving work. The secondary objective is to document how often these different coping strategies are used as well as their correlation with burnout in terms of how often participants feel emotionally exhausted from their work. A mixed methods design was used. The qualitative component, using face to face interviews, allows us to explore the coping strategies that physicians actually use in response to their work-related stress and facilitates the construction of survey items that better represent the coping strategies physicians typically use. The quantitative component, using a survey questionnaire, allows us to document how often physicians use these coping strategies based on a larger, more representative sample, as well as conduct statistical analyses to explore the relationships between the coping strategies and physicians' feelings of emotional exhaustion.

## Methods

### Study design

**Figure 1:**
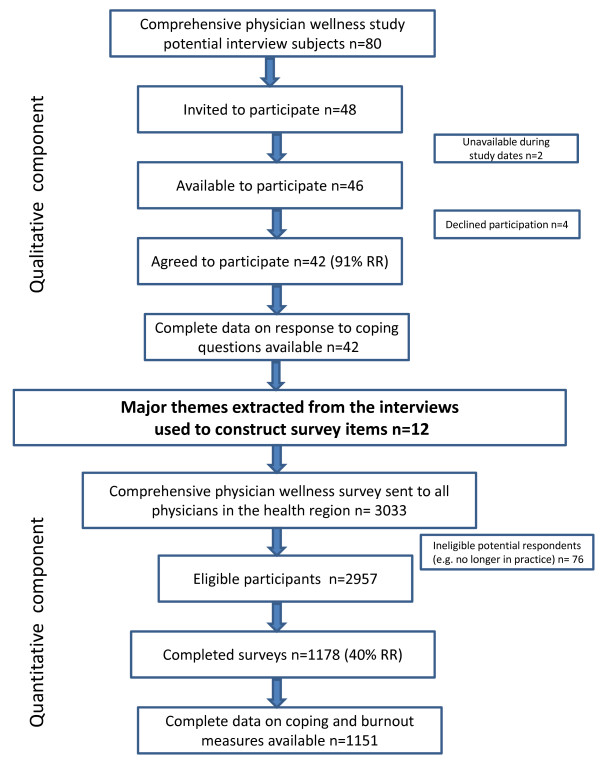
**Mixed methods design study flow chart**.

Figure 1 outlines the flow of the mixed methods study design. The data analyzed for this paper stems from a mixed methods study of physician wellness. During the qualitative component of the study, we conducted interviews to explore physicians' 1) perceptions of the link between their wellness and quality of patient care; 2) their work activities, workload and work time; 3) what they enjoy about their work; 4) their stresses; and 5) their coping strategies and support systems. The quantitative component included a questionnaire constructed from the major themes extracted from the interviews. It was sent to all the physicians in the health region in order to quantify the findings from the qualitative component using a larger, more representative sample, and to explore the correlations between the various factors related to physician wellness. The qualitative findings reported in this paper are based on self reported coping strategies collected through open ended interviews with 42 physicians representing diverse medical specialties and settings (91% response rate). The major themes relating to coping strategies extracted from the qualitative interviews were used to construct 12 survey items that were included in the comprehensive quantitative questionnaire. Questionnaires were sent to all eligible physicians in the health region with 1178 completed surveys (40% response rate.) Questionnaire items were used to measure how often physicians use the various coping strategies. Feelings of burnout were also measured by 5 items of the Emotional Exhaustion subscale from the revised Maslach Burnout Inventory [11].

### Setting

The study was conducted within a single large health region in Western Canada that administers 43 health care centers and 12 acute care sites (including a major university teaching hospital, 3 additional urban hospitals, and five rural hospitals) and employs almost 3000 physicians. Interviews were conducted between September 2006 and July 2007. The surveys were mailed out in March of 2008.

### Participants

For the qualitative component of the study, a convenience sample of roughly 40 physicians was achieved in the following manner. The research team generated a sampling frame of 80 potential participants. We used a non-probability quota sampling to select participants from different medical specialties within the health region. The number of participants selected per specialty was calculated to reflect the proportions within the health region. Within each specialty we also endeavored to select physicians who varied by sex, life stage and work site (e.g. private clinic, hospital clinic, community clinic, hospital unit), identifying first and alternative contacts in each category. From the sampling frame of 80 potential participants, some were known to the researchers as colleagues and some were randomly chosen from a central listing of physicians in the health region. We then invited 48 physicians to participate. Four refused and two were unavailable. Of the 46 who were available, 42 agreed to participate (91% response rate). For the quantitative component of the study, all physicians in the health region were sent a questionnaire. We received 1178 surveys from an eligible 2957 (40% response rate). Complete data on coping and emotional exhaustion were available for 1151 respondents. Table 1 demonstrates how the participant samples for both components are proportionally representative of the physicians in the health region in terms of their medical specialty.

**Table 1:** Breakdown of physician specialty for qualitative component health region versus interview sample (June 2006) and quantitative component health region versus survey sample (June 2008)

	Qualitative component (interviews) June 2006		Quantitative component (survey) June 2008	
*Specialty*	***Health Region***^a^ *N(%)*	*Sample* *N(%)*	***Health Region***^a^ *N(%)*	*Sample* *N(%)*
Anesthesiology	138 (6%)	3 (7%)	145 (6%)	73(6%)
Cardiology	71 (3%)	2 (4%)		
Clinical Neurology	60 (3%)	1 (2%)	72 (3%)	18 (2%)
Critical Care	43 (2%)	1 (2%)		
Diagnostic Imaging	83 (4%)	2 (4%)	83 (4%)	22 (2%)
Emergency	81 (4%)	2 (4%)	87 (3%)	58 (5%)
Family	695 (31%)	11 (26%)	772 (31%)	407 (33%)
Internal Medicine^b^	271 (12%)	6 (14%)	447 (18%)	199 (17%)
Obstetrics/Gynecology	60 (3%)	2 (4%)	68 (3%)	24 (2%)
Pathology	60 (3%)	1 (2%)	66 (3%)	26 (2%)
Pediatrics	174 (8%)	4 (9%)	198 (8%)	78 (7%)
Psychiatry	123 (6%)	2 (4%)	123 (5%)	73 (6%)
Rural^c^	142 (6%)	4 (9%)	181 (7%)	82 (7%)
Surgery	206 (9%)	4 (9%)	220 (9%)	117 (10%)
Other				83 (7%)
**TOTAL**	**2207 (100%)**	**42 (100%)**	**2488 (100%)**	**1178 (100%)**

^a ^Based on Department and division counts for the Health Region June 2006 and 2008

^b ^Includes cardiology and critical care in 2008

^c ^Included one anesthesiologist and three family physicians for 2006. In our 2008 survey, we asked physicians if they practiced in a rural area in a separate question about location of practice. The 82 rural physicians in our sample are classified both in their specialty area and as rural physicians.

### Data sources/measurement

During the interviews, physicians were asked to describe their day-to-day work experiences as well as the most stressful aspects of their work. Following this, we explored the coping strategies physicians use by asking: "What do you do after a bad or hard day at work? How do you cope while you are at work and what do you do when you leave work?", the answers to which are the focus of this paper. If needed, prompts were used to elicit more detailed information regarding the coping strategies participants' identified, as well as where they were used (e.g., at work or at home) and whether their work stress is limited to the workplace or extends beyond the workplace such that they use coping strategies outside of work. In the questionnaire, we measured the frequency of use of the most common coping strategies that were identified from the interviews with 7 items related to coping with work stress while at work, and 5 items related to coping with work stress after leaving work. Several of the work based items were adapted from existing scales [12-14]. We prefaced the survey items with the following statement: "In our interviews with physicians, we found that they often use different strategies for coping with the stresses of their work. Thinking about the ways you deal with stress, how often do you do each of the following?" The 7 items that tap coping strategies that physicians use at work are as follows: I make a plan of action and work through it; I go on as if nothing has happened; I keep it to myself; I concentrate on what I have to do next; I take a time out; I talk it over with colleagues; and I use humor to lighten the situation. The 5 items that reflect those strategies used outside of work are as follows: I find time to exercise; I set aside some quiet time for myself outside of work; I spend time with my family outside of work; I leave work at work; and I talk it over with my spouse. The response categories included never; not very often; sometimes; often; and most of the time. In the questionnaire, we measure burnout using 5 items from the Emotional Exhaustion subscale from the Maslach Burnout Inventory (MBI) (General Survey). We used Barnett et al's [11] revised version of the MBI that directly asks about the respondent's feelings and that includes mutually exclusive response categories. We limited our operationalization of burnout to Emotional Exhaustion due to space limitations in the survey. Physicians are known to be particularly prone to low response rates [15-17] and the survey needed to be brief in order to maximize the response rate. Since the survey covered several different topics, a decision was made to cover a broader range of variables using fewer items, rather than including a limited number of exhaustive "gold standard" measures [16]. We chose to include only the 5 items tapping the emotional exhaustion dimension of burnout, which appears to be the best understood and critical in understanding the burnout process [18]. Respondents were asked to indicate how often they experience the following: I feel emotionally drained from my work; I feel used up at the end of the workday; I feel tired when I get up and have to face another day on the job; I feel that working all day is really a strain for me; and I feel burned out from my work. The response categories included never (coded 1); not very often (coded 2); sometimes (coded 3); often (coded 4); and most of the time (coded 5). A mean frequency of emotional exhaustion was computed by summing the score of the 5 items and dividing by 5 (the number of items). A higher score indicates more frequently experiencing feelings of emotional exhaustion. We conducted confirmatory factor analysis of the 5 items by means of maximum likelihood estimation and all 5 items have acceptable factor loadings (all greater than .8) on a single factor and the inter-item reliability (alpha = .90) is comparable to those reported for the revised and original Emotional Exhaustion subscales [11].

### Bias

A quota sampling strategy was used to generate the sampling frame of 80 potential interview candidates. However, there was potential bias in that the research team included four locally practicing physicians who collectively knew many of the potential participants. For the quantitative component of the study, physicians who responded to the questionnaire may have been more interested in physician wellness or had more time to complete the survey.

### Study size

For the qualitative component, interviews were conducted with 42 physicians from a single health region. For the quantitative component, we targeted all practicing physicians within the same health region (n = 2957) and 1178 returned completed questionnaires.

### Analytic method (qualitative component) and statistical methods (quantitative component)

**Figure 2:**
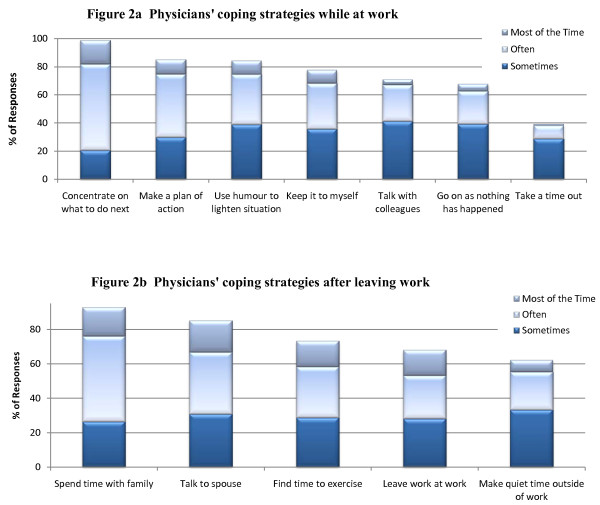
**Frequency of use of physicians' coping strategies (n = 1151)**.

For the qualitative component, the authors and the research assistant used an inductive strategy through open and selective coding to derive the predominant themes reflected in the interview transcripts. For the quantitative component, first we calculated the frequency results for the coping strategies. In Figure 2, we focus on the extent to which the coping strategies are regularly used by reporting whether respondents use that strategy sometimes, often or most of the time. To determine the relationship between each of the coping strategies and how often physicians feel emotionally exhausted, zero-order correlations were used. The correlation indicates the direction and magnitude of the relationship between each pair of variables. A statistically significant positive correlation means that the more frequently physicians use that coping strategy, the more often they experience emotional exhaustion, or symptoms of burnout. A statistically significant negative correlation means that the more frequently physicians use that coping strategy, the less often they experience emotional exhaustion.

Ethics approval for this study was obtained from the Conjoint Health Ethics Review Board of the University of Calgary.

### Role of the funding source

Support for this research was provided by a Research Grant from the Alberta Heritage Foundation for Medical Research's (AHFMR) Health Research Fund and financial and in-kind support from the former Calgary Health Region (CHR) now Alberta Health Services. The funding sources had no role in the study's design, conduct or reporting.

## Results

### Qualitative component (Interviews)

#### Participants

The sample of physicians interviewed included 19 women (45%) and 23 men (55%). Most of the participants (86%) were married at the time of the study and most of the participants (90%) were parents. On average, the physicians in this study were 47 years of age and had practiced medicine for 15 years but the sample varied greatly. Most of the participants worked full time (79%), most take call (81%) and almost half (43%) were involved in a group practice. The sample reported working, on average, approximately 56 hours per week when not on call, although this varied from 30 to 90 hours. Also see Table 1 for participants' medical specialties.

#### Themes

Five major themes reflecting the coping strategies that physicians use while at work were identified from the interview data. These include: Working through or simply dealing with the stress at work; talking with co-workers; taking a time out; using humor; and ignoring or denying the stress. The most popular coping response is working through or simply dealing with the stress at work. Participants often described this strategy as one where they "get the job done", "just power through it", "soldier on", or "get on with it". Some of the participants also seem to recognize that this may not be a healthy coping strategy. For example, they refer to it as "kind of sick", or "it's not good". The following quote illustrates this theme:

... I don't think I do much to deal with it at work. I just keep, keep, keep on going and I'm somewhat obsessive compulsive I think. I know I am and I think that most doctors are quite frankly. So part of what relieves my stress is getting everything done. And if I have a dictation that's hanging over my head like I did today, that stresses me, so I try and get that kind of stuff done... I relieve stress by continuing to work. I mean, it's kind of sick [laughs].

The second most popular coping strategy is talking to co-workers, where the physicians received both emotional and informational support (e.g. discussing a case) from their colleagues. Many physicians also described taking a time out, which might involve stepping outside for a breath of fresh air, going for a tea or coffee break, or closing the door to their office for a bit of quiet time. Using humor, joking with staff, and laughing about stressful incidences are also described coping strategies.

Well, today I had some stress because of delays... I went and stood outside the door and took a few breaths of the nice, sunny air. Put my jacket back on, went back, felt much better [laugh]. I might um have a little joke with the staff or have a cup of tea. That usually works.

Lastly, participants indicated that they denied, ignored or put stress aside while they are at work using descriptors like "putting stress on the back burner", and "just block it out".

Usually denial. I just put it on the back burner and just go on 'cause you know, if you get too stressed out then you can't function and so I usually just suppress it [stress].

For coping with work related stress after leaving work, the major themes identified are the following: Exercising; having quiet time, talking to spouse, spending time with family and leaving work at work. Other minor themes include doing more work at home, talking with others, and having a drink of alcohol. Most physicians regularly use exercise as a coping strategy in response to work related stress, indicating that they often run, go to the gym, or cycle.

I like to swim and exercise. I mean that's a pretty good outlet for me. I probably depend on, maybe depend is a strong word, I like to have a drink at the end of the day if I'm stressed for sure. Um, that seems kind of negative, that's true though.

Some physicians use more quiet coping strategies like spending time alone with the door closed at home, watching television, reading, or doing a "mindless" activity.

When I go home, mostly I would just do something that's non-thinking... I just turn off into some kind of mindless, or just read a book or watch mindless TV, or go out for dinner.

Participants also report that they talk with their spouse after a difficult day at work, often over dinner or when they are out for a walk together, or spend time with extended family in order to relieve work related stress.

... I usually go for a walk with my wife and the dog. You know, just kind of talk, um you know, I love watching my kids play sports so I go to their games.

Many also describe just "leaving work at work" and not taking on work tasks at home such as cleaning up their email inbox.

### Quantitative component (Survey)

#### Participants

Of the physicians who completed the questionnaire items relevant to this analysis, 665 (58%) were men and 486 (42%) were women. At the time of the study, on average, our respondents were 49 years of age (range = 27-89 years) and had practiced medicine for about 18 years. Most of the participants take call (77%) and half (50%) were involved in a group practice. The sample reported working, on average, approximately 50 hours per week when not on call and when they were involved in mainly patient-care related duties, although this varied greatly. Also see Table 1 for medical specialties of the physicians who completed our survey.

#### Frequency of use of coping strategies

Figure 2a shows the percentage of physicians who report sometimes, often or most of the time using the different coping strategies for work related stress while at work. The most popular coping strategy is concentrating on what they have to do next, used by virtually all of the respondents at least sometimes. More than half of the respondents (61%) reported that they use this strategy often and 17% reported they use it most of the time. About 85% of respondents make a plan of action and work through it or use humor to lighten the situation at least sometimes. 78% keep stress to themselves, and about 70% talk with colleagues or go on as if nothing has happened at least sometimes. Only 38% take a time out, with most of these (29%) using this coping strategy only sometimes. Figure 2b shows the percentage of physicians who report sometimes, often or most of the time using the different coping strategies for work related stress after they leave work. The two most popular strategies appear to involve their families. Virtually all of the physicians report that they spend time with their family after a difficult day at work with half (49%) reporting they use this strategy often and 17% reporting they use it most of the time. About 80% of respondents talk with their spouse, 73% find time to exercise, 68% leave work at work, and 62% make quiet time outside of work at least sometimes.

#### Coping strategies and how they relate to emotional exhaustion

Table 2 shows how the coping strategies relate to how often physicians feel emotionally exhausted or burned out from their work. Of the 7 key coping strategies that physicians use to deal with work stress at work, 4 were significantly negatively correlated (p ≤ 0.001), but 3 were significantly positively correlated (p ≤ 0.001) with emotional exhaustion. Those that were associated with a lower frequency of emotional exhaustion include taking a time out (r = -.18), using humor to lighten the situation (r = -.11), talking it over with colleagues (r = -.11), and making a plan of action (r = -.10). Recall from figure 2a that very few physicians take a time out from a stressful situation at work and that making a plan of action is the second most commonly used coping strategy. The three coping strategies associated with a higher frequency of emotional exhaustion include keeping stress to oneself (r = .23), concentrating on what to do next (r = .16), and going on as if nothing happened (r = .07). Keeping stress to oneself, the coping strategy that is most highly correlated with emotional exhaustion, is also used very often as is concentrating on what to do next, the most popular strategy used by the physicians in this study. All 5 key coping strategies that physicians use to deal with work stress after they leave work are significantly negatively correlated with emotional exhaustion (p ≤ 0.001) including, in order of most to least correlated, setting aside quiet time outside of work (r = -.22), finding time to exercise (r = -.21), spending time with family outside of work (r = -.19), leaving work at work (r = -.17), and talking about stress with their spouse (r = -.06). Recall from figure 2b that the strategies involving family members were the most popular.

**Table 2:** Physicians' coping strategies and how they relate to emotional exhaustion

Physicians' coping strategies while at work	
*Coping strategies that are correlated with a lower frequency of emotional exhaustion*	*Coping strategies that are correlated with a higher frequency of emotional exhaustion*
Take a time out (r = -.18; p < .0001)	Keep stress to myself (r = .23; p < .0001)
Use humor to lighten the situation (r = -.11; p < .0001)	Concentrate on what to do next (r = .16; p < .0001)
Talk it over with colleagues (r = -.11; p < .0001)	Go on as if nothing happened (r = .07; p < .0001)
Make a plan of action (r = -.10; p = .001)	

**Physicians' coping strategies after leaving work**	
***Coping strategies that are correlated with a lower frequency of emotional exhaustion***	***Coping strategies that are correlated with a higher frequency of emotional exhaustion***

Set aside quiet time outside of work (r = -.22; p < .0001)	
Find time to exercise (r = -.21; p < .0001)	
Spend time with family outside of work (r = -.19; p < .0001)	
Leave work at work (r = -.17; p < .0001)	
Talk about stress with spouse (r = -.06; p = .001)	

## Discussion

During the interviews, physicians described their coping strategies in their own words. From analysis of the interview and questionnaire data, we found that the most common coping strategies used at work involve continuing on with their duties despite the stress, dealing with stress through making a plan of action and/or humor, and talking to co-workers. The 3 coping strategies that appear to reflect denial responses to stress, including the most often used coping strategy of simply concentrating on what to do next, were associated with a higher frequency of emotional exhaustion. The least commonly used strategy, taking a time out, as well as all of the coping strategies used outside of work, were negatively correlated with emotional exhaustion, the most popular being spending time with family, talking to one's spouse and physical exercise.

To our knowledge our study is unique in that the mixed methods design provided the opportunity for the physicians themselves to describe their coping strategies, thus facilitating the construction of a relevant and occupation specific questionnaire. The coping strategies reported by the participants are consistent with categories previously described in the general literature, including problem-focused coping which facilitates completion of work tasks (e.g. make a plan of action), emotional-focused coping that assists in managing the emotional reaction to stressors (e.g. use humor to lighten the situation), and potentially maladaptive coping responses (e.g. ignore or deny stress) [19-21]. Our mixed methods study design also allowed us to document the frequency of use of the various coping strategies in a larger, more representative sample and to explore the association between the coping strategies and physicians' feelings of emotional exhaustion. Many of the physicians in our study often use coping strategies that are associated with less frequent feelings of emotional exhaustion, but coping strategies positively correlated with frequency of emotional exhaustion are also often used.

The 3 strategies that were correlated with higher frequencies of emotional exhaustion were those that do not involve actually dealing with the source of stress in a constructive manner (concentrate on what to do next, keep it to myself, go on as if nothing happened). To some degree, these coping responses are consistent with those described as denial or restraint coping in the general literature, that is, waiting for a more appropriate opportunity to react [19-21]. Continuing on with their work despite the stress may reflect a pragmatic approach that enables the physicians to function despite the significant stressors that are part of their day-to-day work environment. Although these coping strategies may temporarily resolve the stressful situation or reduce the amount of work that is stressful, they were associated with more frequent feelings of emotional exhaustion in our study and may be viewed as maladaptive or even harmful. Prior studies of medical and surgical trainees have also shown associations between maladaptive coping strategies and high stress and long work hours, [22] as well as poor virtual laparoscopic surgery performance [23].

In our study, talking with colleagues was a relatively commonly used coping strategy correlated with a lower frequency of emotional exhaustion. The physicians in our study described how this interaction provides an emotional source of support where physicians feel that they are not alone in their experiences, and at times also provides informational support (e.g. dialogue about patient care issues) that helps reduce stress by problem focused coping. The benefit of this coping strategy has been previously shown in a study of internal medicine physicians that demonstrated how collegial support was directly related to physician well being and also buffered the negative effects of work demands [24]. In our study, the emotional focused coping strategies that physicians use outside of work, such as spending time with family and talking with spouse, as well as leisure activities, such as exercise and quiet time, were associated with less frequent reports of emotional exhaustion. This is consistent with the general psychology literature where it has been shown that time away from work, switching off mentally and enjoying a psychological detachment from work during off-job time, and other leisure activities that allow recovery in between work periods result in positive benefits for workers [25-29]. In another example, Sargent et al reported that for physicians, being a parent and spending time with a spouse are protective factors against burnout [2], suggesting that family life may also serve to detract from work stress.

Limitations of the qualitative component of this study include the potential selection bias for interviewed physicians in that the research team generating the initial list of possible participants included locally practicing physicians who knew many of the physicians within the health region. For the quantitative component, there may be a survey response bias in that physicians who responded may be more interested in physician wellness or have more time to complete the survey. In addition, while this study reports significant correlations between certain coping strategies and feelings of emotional exhaustion, it does not prove causation, nor does it explore the association between a particular stress and the coping response. Certain coping responses may be more beneficial for one type of stressor (e.g. workload) and detrimental for another (e.g. difficult interpersonal interactions). Lastly, although there was broad and proportional representation of physicians throughout all the different types of medical practice in the interviews and survey, this study was conducted within a single health region and the results may not be generalizable. Future research may include longitudinal studies to see if the associations between the different coping strategies and emotional exhaustion noted in our study are sustained over the long term.

## Conclusions

In summary, the physicians in our study described the various coping strategies that they use to deal with their work stress, and reported how often they use them. Not all of the coping strategies are created equal as some were used more often than others and many were associated with a higher frequency of emotional exhaustion from one's work. If further research determines that this correlation indicates causation, we could educate physicians about the effectiveness of their coping strategies, and integrate the beneficial strategies into practical stress reduction interventions.

## Competing interests

The authors declare that they have no competing interests.

## Authors' contributions

Both JL and JW contributed to the conception and design of the study, acquisition of data and interpretation of data. JW was primarily responsible for data analysis. Both authors were involved in drafting the manuscript and revising it critically for important intellectual content and have given final approval of the version to be published. We wish to acknowledge Alyssa Jovanovic's invaluable assistance in transcribing and analyzing the interview data.

## Pre-publication history

The pre-publication history for this paper can be accessed here:

http://www.biomedcentral.com/1472-6963/10/208/prepub
